# Curtailment of Civil Liberties and Subjective Life Satisfaction

**DOI:** 10.1007/s10902-021-00491-1

**Published:** 2021-12-22

**Authors:** Lisa Windsteiger, Michael Ahlheim, Kai A. Konrad

**Affiliations:** 1 Max Planck Institute for Tax Law and Public Finance, Marstallplatz 1, 80539 Munich, Germany; 2 Institute of Economics (520F), University of Hohenheim, 70599 Stuttgart, Germany

**Keywords:** Survey experiment, Covid-19, Life satisfaction, Reactance

## Abstract

This analysis focuses on the lockdown measures in the context of the Covid-19 crisis in Spring 2020 in Germany. In a randomized survey experiment, respondents were asked to evaluate their current life satisfaction after being provided with varying degrees of information about the lethality of Covid-19. We use reactance as a measure of the intensity of a preference for freedom to explain the variation in the observed subjective life satisfaction loss. Our results suggest that it is not high reactance alone that is associated with large losses of life satisfaction due to the curtailment of liberties. The satisfaction loss occurs in particular in combination with receiving information about the (previously overestimated) lethality of Covid-19.

**Supplementary Information:**

The online version contains supplementary material available at 10.1007/s10902-021-00491-1.

## Introduction

How much does personal freedom matter for life satisfaction? In Spring 2020, as a regulatory response to the Covid-19 pandemic, basic freedoms were severely restricted in many countries. These included the right to move freely, freedom of travel, freedom of assembly, rights to engage in one’s own business and the right to meet other people, form groups and engage in social interaction. The policy intervention was triggered by an exogenous event (the pandemic), and thus provides us with a unique opportunity to assess the direct well-being costs (being agitated, angry, or feeling hampered in one’s freedom of action) and indirect well-being effects (e.g. consequences of restricted economic freedom). In our analysis, we want to assess what determines the direct costs. In particular, we are interested in how individuals’ life satisfaction losses depend on their aversion to rules and restrictions that threaten or eliminate some of their behavioral freedoms, and whether these losses depend on information about Covid-19 lethality (and thus on how “justified" the restrictions are perceived to be).

To measure subjective well-being we use the concept of self-assessed life satisfaction and its change between pre-crisis and crisis times in 2020.^1^ The Covid-19 pandemic crisis and the policy reactions to it might have caused a reduction in life satisfaction for many reasons apart from the freedom-restricting measures per se. However, we focus on the effect of randomized information treatments about the lethality of Covid-19 paired with individual heterogeneity in respondents’ reactance—a personal trait that can be regarded as a measure of the general aversion to rules and restrictions imposed from outside. This allows us to make statistical inferences about the contribution of the restriction of civil liberties to the overall reduction in life satisfaction, depending on respondents’ perception of the danger emanating from the virus and their aversion to imposed restrictions.

The relationship between measures of freedom and measures of life satisfaction or subjective well-being has attracted considerable attention by researchers.^2^ While there is no perfect consensus and much debate about possible channels, the majority view is that there is a positive correlation or potentially even causation. These studies overwhelmingly exploit heterogeneity between countries and/or over a series of years. Our analysis is methodologically different in that we study an exogenous event that led to a sudden and serious curtailment of behavioral freedoms within a given overall political and institutional arrangement and with given societal norms. We also make use of experimental variation caused by three information treatments concerning the lethality of Covid-19.

For the measurement of freedom preferences we use a concept that has been developed in psychology: a personal trait that could loosely be paraphrased as describing the intensity by which individuals are averse to regulations or prohibitions curtailing their freedom and autonomy. Individual heterogeneity in the strength of this trait is known and measured as * reactance * (see Brehm, 1966, and a review by Miron & Brehm, 2006). The well-established methodology to measure the strength of such aversion is the *Hong Psychological Reactance Scale* (see Hong & Page, 1989). The reactance scale is generated from a set of 14 questions. A factor analysis is used to see how individual reactance is best described by a weighted average of the various answers in the sample. This index number is used as the measure of the individual respondent’s strength of reactance. There is an ongoing discussion in the literature concerning a potential decomposition of the reactance trait into two or more factors (see e.g. Hong & Faedda, 1996; Brown et al., 2011; De las Cueavas et al., 2014; Yost & Finney, 2018) suggesting that one, two or four factors might be appropriate. The factor analysis of the answers to the Hong reactance scale in our survey suggests that a single dimension is suitable to measure reactance, and each respondent’s answers to the reactance questions are transformed and mapped into a single value of a reactance index (see supplementary material). Together with other control variables, this score is used to estimate or explain differences in the change in subjective life satisfaction upon receiving information about estimated Covid-19 lethality.

Our results suggest that both psychological reactance and receiving information about Covid-19 lethality lead to a higher loss in subjective life satisfaction from the pandemic crisis and the ensuing lockdown measures. But it is in particular the combination of the two that triggers an additional decline. We find that respondents who are averse to restrictions imposed from outside, when informed that the virus is not as dangerous as they initially thought, suffer more from the lockdown measures than our uninformed control group. The loss in life satisfaction thus seems to depend on what respondents know about the situation in which such freedom restricting measures are taken and whether these restrictions are ‘justified’ in their eyes.

Our results also provide insights into how the pandemic crisis affects life satisfaction in general, and which factors drive the change in life satisfaction. Related studies have been conducted for other crises (see e.g. Deaton, 2012 for the impact of the financial crisis on subjective life satisfaction in the US, and Tonzer , 2019 for the impact of the financial crisis 2007–2012, focusing on employment status and macroeconomic institutions as explanatory factors). Several factors might explain a reduction in life satisfaction during the 2020 pandemic crisis. Our results shed some light on the role of employment security and the existential risk of the lockdown for self-employed individuals.

## The Survey Experiment

**Participants ** The data for our study was collected in an internet-based survey of a sample of 4000 individuals. The survey was designed and programmed by the authors via Qualtrics and administered by the survey company Respondi between April 10 and April 20, 2020, in a time period when the restrictions on basic freedom rights in Germany had been in place for several weeks already. Most respondents replied prior to clear policy decisions about whether these restrictions would be continued for a further period or for how long.

Respondi allows researchers to influence the self-selection of respondents into the sample for the study by setting quotas for gender, age, marital status and household income. The sample can be considered as representative in terms of these criteria. Participants were compensated by a flat rate.

**Questionnaire ** The main outcome variable of interest for this study was the change in assessed life satisfaction between the beginning of the year 2020 and the date of the survey in April 2020.^3^ We also elicited a number of other outcome variables which might be studied in future work (see the questionnaire in the supplementary material). The main explanatory variable we focus on is the respondents’ reactance as a measure of aversion to prescriptions and liberty restraints which is extracted from 14 questions following Hong and Page (1989). We aim to exploit the interpersonal heterogeneity in the strength of this index to assess the direct impact of the curtailment of choice freedom on the size of the loss in life satisfaction for individuals with different strength of reactance. The control variables included in our regressions are: equivalized household income (using the OECD method, i.e. dividing household income by the weighted number of household members, where the first adult counts as 1, every additional grown-up as 0.5 and every child as 0.3), age, gender, marital status, number of grown-ups in the household, number of children in the household, equivalized living space (again using the OECD equivalization method), a dummy for whether the respondent has been infected with Covid-19, a dummy for whether the respondent thinks she can protect herself from infection also after the lockdown measures are lifted, a dummy for whether the respondent works in a sector with high work load at the moment (health, education, groceries or police), locus of control (an index constructed from four questions via factor analysis, a higher value means outer locus of control), a dummy for whether the job is negatively affected (lost job or on short term work), a dummy for whether the respondent has his/her own company or shop, the 7-day-incidence rate of Covid-19 cases in respondents’ residential region, and we also control for the state in which the respondent lives (not shown in regression tables).

**Information treatments ** The programming tool Qualtrics was used to randomly partition the full sample of respondents into three separate information treatments. These treatments differed in the information about the reported lethality of Covid-19 in the Wuhan area. The base treatment $T_{0}$ provided no such information. Treatment $T_{1}$ provided information about the average lethality of Covid-19 in the Wuhan area, and treatment $T_{2}$ provided information about the distribution of lethality across the age distribution, for 10-year age brackets of the population. The information was taken from an analysis of the Covid-19 outbreak in Hubei province, China (see Tan et al., 2020), which was one of the first systematic studies on Covid-19. A detailed description of our information treatments is provided in the supplementary material.

How reactance affects the loss in life satisfaction might depend on whether the respondent thinks that the prescriptions and the curtailment of freedoms serve a good cause or not. Direct measures of such assessments are endogenous to reactance itself, however. One factor that might affect people’s assessment of the restrictive measures and their necessity is the (perceived) lethality of the virus. Our randomized information treatments at the beginning of the survey serve the purpose of experimentally manipulating those lethality beliefs. By randomly providing information about average Covid-19 lethality (treatment $T_{1}$) or age-dependent lethality ($T_{2}$) to some respondents, we can evaluate how this information affects their (loss in) life satisfaction compared to the control group $T_{0}$ who does not receive such information.

To check whether our information treatments were successful in shifting beliefs about Covid-19 lethality, we elicit beliefs about lethality rates from all participants at the end of our survey. Specifically, we ask them to assess average lethality of Covid-19 (in the Wuhan area), lethality among the young (20 to 29 year old) and lethality among the old (+80 years old). Participants in our base treatment $T_{0}$ got no information on lethality, thus we consider their “posterior” lethality beliefs as a proxy for untreated prior lethality beliefs in our whole sample. By comparing the posterior beliefs in treatment group $T_{1}$ and $T_{2}$ to those in $T_{0}$ we can assess how lethality beliefs were affected by our information treatments on average. Furthermore, analyzing how close the posterior beliefs in $T_{1}$ and $T_{2}$ are to the numbers we provided in the information treatments at the beginning of the survey tells us how much attention participants paid to our treatments and thus how effective they were (in terms of influencing lethality beliefs and thus the assessed danger of Covid-19).

**Change rather than level effects ** Within psychology, the study of the impact of psychological traits on life satisfaction is well established and constitutes a broad area of research (see e.g. De Neve et al., 1998; Steel et al., 2008 for meta-analyses about the correlations between psychological traits and levels of subjective life satisfaction for a large number of traits).

The main outcome variable we study is not the level of life satisfaction. Similar to Arampatzi et al. (2020) in a different context, we focus on the *change *in self-assessed life satisfaction and explore the effect of our information treatments on this variable, as well as the explanatory power of psychological traits and socioeconomic characteristics. The change in life satisfaction is measured as $D_{i}\equiv w_{oi}-w_{ni}$ : the remembered life satisfaction of a person *i* at the beginning of 2020 $(w_{oi})$ minus the assessed life satisfaction at the time of the survey $(w_{ni})$ in April 2020 (old–new, such that a positive value means that life satisfaction has declined). We expect that this change is a loss on average, due to the crisis, implying that life satisfaction has declined for many people from the beginning of 2020 to April 2020.

We look at the curtailment of freedom rights and ask: is the implied loss in life satisfaction larger for persons with a stronger aversion to restrictions to free choice? The information treatments might also affect assessed life satisfaction: Specifically, individuals with strong aversion to regulations and prohibitions might lose more life satisfaction if they learn that these prohibitions were imposed in an environment with much lower lethality than they initially believed. Individuals’ losses in life satisfaction might not depend on their aversion to prohibitions as such, but on their beliefs about the conditions that motivate such prohibitions.

## Results

**Fig. 1:**
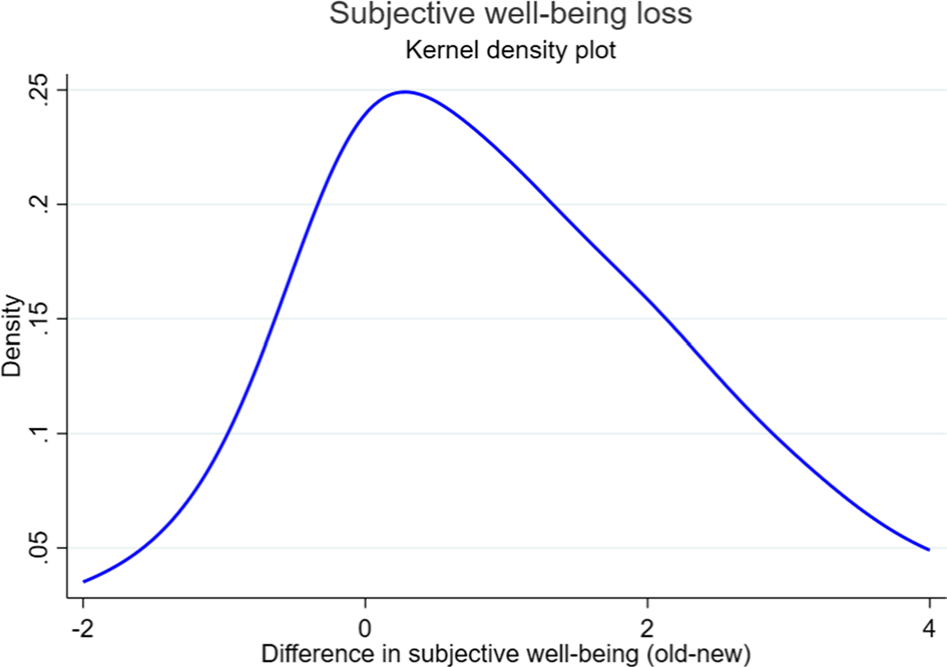
Kernel density plot of the loss in life satisfaction in the control group

Our results show a large loss in life satisfaction during the crisis on average, compared to the remembered pre-crisis values. The average loss in subjective life satisfaction, measured on the standard life satisfaction scale from 1 (entirely not satisfied with one’s life) to 10 (entirely satisfied with one’s life), is 1.1 in our control group $T_{0}$ (who received no information treatment) and distributed as shown in Fig. 1.

Subjects’ remembered level of life satisfaction at the beginning of the year, which serves as a reference value here to be compared with the stated actual level of life satisfaction in April, might be imprecise. There is a whole literature on remembered utility and the validity problems it might cause (see e.g. Kahneman & Riis, 2005; Kahneman, 2011; Mogilner & Norton, 2019). Remembered life satisfaction at the beginning of the year $w_{oi}$ might thus systematically differ from the assessed value if asked at that very time in January. However, comparing the remembered life satisfaction value with the GSOEP data for Germany for May/June 2019, which is 7.15 (Grimm & Raffelhüschen, 2019), and taking into account that subjective life satisfaction generally tends to be lowest in Winter/January (see Maennig et al., 2014) provides an indication that the drop in life satisfaction since January 2020 that we find in our data might actually even be a conservative proxy for the true loss in happiness people experienced during that time period due to the Corona crisis.

More importantly, a systematic bias in the level of $w_{oi}$ due to remembered assessment is not a problem for our main analysis. Our emphasis is not on the average aggregate loss and its measurement: we estimate and try to explain the individual *differences* in the loss in life satisfaction, using our randomized information treatments, as well as reactance as the main indicator for aversion to the curtailment of choice freedoms and prohibitions. A possible systematic level bias for the *remembered* assessment of life satisfaction at the beginning of the year drops out if we consider the role of heterogeneity in the explanatory factors for the measured differences in the loss in life satisfaction.

In our main specification, we estimate the following ordinary least squares (OLS) regression model:

1 $$\begin{aligned} D_{i}=\alpha _{i}+\beta c_{i} + \gamma r_{i} + \delta ^{\prime }_{i}X_{i} + \varepsilon _{i}\quad , \end{aligned}$$

where $D_{i}$ denotes the individual loss in subjective life satisfaction, $c_{i}$ is a categorical variable uniquely identifying each of our treatments with Treatment $T_{0}$ as a baseline, $r_{i}$ denotes the individual reactance index and $X_{i}$ is a vector containing control variables such as respondent *i*’s (remembered) level of life satisfaction at the beginning of the year (stated at the beginning of the survey)^4^ and her sociodemographic characteristics. $\varepsilon _{i}$ is a random error component. Standard errors are clustered at regional (’Landkreis’) level. As a robustness check for our results, we also estimate an ordered probit model.^5^ The qualitative nature of our results does not change in the ordered probit specification. For ease of interpretation, we will thus refer to the OLS estimation results in the main text, but we also report the ordered probit results in the supplementary material.

**Table 1:** OLS regression of the loss in subjective life satisfaction on reactance and controls

Variables	(1)	(2)	(3)	(4)	(5)
	Wholesample	$T_{0}$	$T_{1}$	$T_{2}$	Interactions
	swb loss	swb loss	swb loss	swb loss	swb loss
Reactance	0.18***	0.08	0.14**	0.32***	0.09
	(0.03)	(0.06)	(0.06)	(0.06)	(0.06)
1. Treatment	– 0.01				– 0.01
	(0.07)				(0.07)
2. Treatment	0.14*				0.13*
	(0.07)				(0.07)
1. Treatment*reactance					0.05
					(0.08)
2. Treatment*reactance					0.22***
					(0.08)
swb old	0.57***	0.59***	0.58***	0.56***	0.57***
	(0.02)	(0.03)	(0.03)	(0.03)	(0.02)
hhincome	– 0.00	– 0.00*	0.00	0.00	– 0.00
	(0.00)	(0.00)	(0.00)	(0.00)	(0.00)
Age	0.00	– 0.00	0.00	0.01*	0.00
	(0.00)	(0.00)	(0.00)	(0.00)	(0.00)
Female	0.40***	0.22**	0.34***	0.64***	0.40***
	(0.06)	(0.10)	(0.09)	(0.10)	(0.06)
Single	0.19***	0.03	0.21*	0.30**	0.19***
	(0.07)	(0.12)	(0.12)	(0.12)	(0.07)
nrgups	– 0.00	– 0.05	– 0.00	0.06	– 0.00
	(0.03)	(0.06)	(0.04)	(0.07)	(0.03)
nrkids	0.01	0.11*	– 0.06	– 0.06	0.01
	(0.04)	(0.06)	(0.09)	(0.08)	(0.04)
Living space	– 0.00**	– 0.00	– 0.00	– 0.00	– 0.00**
	(0.00)	(0.00)	(0.00)	(0.00)	(0.00)
Infected	– 0.25	– 0.19	– 0.53	0.05	– 0.25
	(0.29)	(0.57)	(0.43)	(0.40)	(0.29)
Can protect oneself	– 0.04	– 0.23**	– 0.19	0.31**	– 0.03
	(0.08)	(0.12)	(0.13)	(0.13)	(0.08)
Sector	– 0.15**	– 0.13	– 0.18	– 0.09	– 0.15**
	(0.08)	(0.14)	(0.13)	(0.11)	(0.08)
Locus of control	0.52***	0.58***	0.60***	0.42***	0.52***
	(0.04)	(0.07)	(0.08)	(0.07)	(0.04)
Job neg. affected	0.35***	0.28**	0.33**	0.50***	0.35***
	(0.08)	(0.14)	(0.14)	(0.15)	(0.08)
Self-employed	0.75***	0.88**	0.45**	0.96***	0.75***
	(0.16)	(0.36)	(0.22)	(0.22)	(0.16)
Incidence	– 0.00	– 0.00	0.00	0.00	– 0.00
	(0.00)	(0.00)	(0.00)	(0.00)	(0.00)
Constant	– 3.36***	– 2.71***	– 3.14***	– 4.20***	– 3.35***
	(0.25)	(0.44)	(0.42)	(0.46)	(0.24)
Observations	3996	1348	1321	1327	3996
R-squared	0.28	0.30	0.28	0.31	0.28

Specification (1) denotes the regression over the whole sample, (2) (3) and (4) the split sample regressions looking at each treatment group separately, and (5) is the regression over the whole sample including interactions between treatment group and reactance

Control variables included in the regression are: (remembered) life satisfaction at the beginning of the year, equivalized household income, age, gender, marital status, number of grown-ups in the household, number of children in the household, equivalized living space, a dummy for whether the respondent has been infected with Covid-19, a dummy for whether the respondent (thinks she) can take measures to protect herself from infection also once the lockdown is lifted, a dummy for whether the respondent works in a sector with high work load at the moment (health, education, groceries or police), locus of control, a dummy for whether the job is negatively affected, a dummy for whether the respondent has his/her own company or shop, the 7-day-incidence rate of Covid-19 cases in the respondent’s region at the time of the survey, and the state in which the respondent lives (not shown in regression table). Standard errors are clustered at regional (‘Landkreis’) level

Robust standard errors in parentheses

***$$p<0.01$$, **$$p<0.05$$, *$$p<0.1$$

The estimation results for the loss in subjective life satisfaction as the individual respondent’s outcome variable are shown in the first column of Table 1. The reduction in life satisfaction is larger for individuals with higher reactance, i.e. for individuals who have a stronger aversion to the curtailment of choice freedom. The coefficient measuring the effect of reactance on loss in life satisfaction is positive and significant at the 1-% level. This can be interpreted as evidence in line with a theory suggesting that the curtailment of freedom has a stronger direct effect on life satisfaction for individuals who have a higher aversion to regulations. In fact, the size of our coefficient (0.18) suggests that an increase in reactance from the lowest to the highest decile (which, as reactance in our sample turns out to be approximately normally distributed with a standard deviation of 1, amounts to an increase in 2.4 of the reactance index) implies an additional life satisfaction loss of 0.43 points, which is roughly equal to the loss arising from unemployment in previous happiness studies (see Felbermayr et al., 2017). We also find that respondents who receive information treatment $T_{2}$ suffer from higher loss in life satisfaction compared to the control group. The coefficient (0.14) is similar in size to the coefficient on reactance, though only significant at the 10-% level.

Several other variables have a significant effect. Our results suggest that the loss in life satisfaction is larger for employed individuals who fear that they might lose their jobs or who were sent on short-term work ($p < 0.01$), for females ($p < 0.01$), and for entrepreneurs and self-employed individuals ($p < 0.01$)—many of whom might face existential risks due to the lockdown measures. The coefficients of these three variables are large. Fear about job loss increases the loss in life satisfaction by 0.28 in the control group (see column 2 in Table 1, which reports the regression coefficients for the (zero information) control group $T_{0}$). The effect for entrepreneurs or self-employed is even larger at 0.88.

The coefficients of reactance and of the dummy for treatment $T_{2}$ suggest a closer look into the role of the combination of the two. Columns (2)–(4) in Table 1 report the estimates, splitting the whole sample across treatments: the baseline treatment $T_{0}$ (no lethality information provided), the low information treatment $T_{1}$ (information about average lethality provided) and the high information treatment $T_{2}$ (age-cohort dependent lethality information provided). The effect of higher reactance on life satisfaction loss in the overall estimation is driven by information treatment $T_{2}$, where age-cohort dependent lethality information was provided. When restricting attention to treatment group $T_{2}$, the coefficient of reactance is almost twice as high as over the whole sample and highly significant, suggesting that reactance played an important role in lowering respondents’ life satisfaction once they were informed about age-dependent lethality rates of Covid-19. Reactance does also seem to lower life satisfaction in $T_{1}$, though to a smaller extent and the coefficient is only significant at the 5-% level. Very similar results emerge from including interaction terms of reactance with the treatment conditions (see column (5) in Table 1): the life satisfaction loss effect of high reactance occurs in the context of information treatment $T_{2}$ and the estimated coefficient on the interaction term is highly significant.

Our results suggest that it is not high reactance alone that is associated with large losses of life satisfaction due to the curtailment of liberties. The satisfaction loss occurs in particular in the context of information treatment $T_{2}$. The information provided in this treatment might have a differential effect on people depending on their reactance level. A comparison between untreated lethality beliefs (in treatment $T_{0}$) and the data reported about Wuhan indicate that a large majority of respondents in the control group vastly overestimate the lethality risks (see supplementary material). We thus conclude that the information provided in treatment $T_{2}$ has triggered a downward adjustment in treated respondents’ lethality beliefs on average. At the same time, whether the curtailment of fundamental liberties is seen as a legitimate act or as an encroaching and arbitrary act might exactly depend on beliefs about the lethality of the virus, and this distinction might be relevant for whether highly reactant people feel a smaller or a larger loss in life satisfaction from these measures.

To corroborate this intuition, we examine treatment group $T_{1}$ in more detail. In the split sample regression (see column (3) in Table 1) we find that reactance increases the loss in life satisfaction also in $T_{1}$, but the coefficient is only significant at the 5-% level (and the coefficient on the interaction between reactance and $T_{1}$ in column (5) is insignificant). Now we want to examine whether the effect is stronger for respondents who are better able to recall the “correct" average lethality of Covid-19 at the end of the survey. As we provide this information at the beginning of the survey in $T_{1}$ and as we know that “untreated” subjects (in $T_{0}$ ) tend to (vastly) overestimate these rates, we assume that those subjects who state approximately correct beliefs at the end of the survey have revised their lethality estimates (typically from very high values dramatically downward) as a result of the information provided in this treatment. On the other hand, we assume that respondents in $T_{1}$ with posterior beliefs about lethality rates that are much higher than the numbers provided in our information treatment were inattentive or ignored the information for other reasons (maybe because they did not believe in the information we provided), and thus our treatment did not work to affect their beliefs. Our treatment might thus have less effect on those individuals who fail to correctly recall the information we gave them at the beginning—which could explain the overall weak effect we find concerning the combination of reactance and treatment $T_{1}$—but the effect might be stronger for people with posterior beliefs closer to the information we provided. By analyzing the interaction between reactance and respondents’ beliefs about average lethality we can check whether this intuition is supported by our data and reactant people with *correct* beliefs indeed suffer from higher life satisfaction losses than those who did not update their beliefs sufficiently.

The results of the interacted regression are given in Table 2.

**Table 2:** OLS regression of the loss in subjective life satisfaction on reactance, the respondent’s belief about average lethality, the interaction and controls, regression restricted to treatment group (1) $T_{1}$ and (2) $T_{2}$

Variables	(1)	(2)
	$T_{1}$	$T_{2}$
	swb loss	swb loss
Reactance	0.23***	0.34***
	(0.06)	(0.06)
Average lethality belief	– 0.00	– 0.00
	(0.00)	(0.00)
Reactance * av. leth. bel.	– 0.01***	– 0.00
	(0.00)	(0.00)
swb old	0.57***	0.56***
	(0.03)	(0.03)
hhincome	0.00	0.00
	(0.00)	(0.00)
Age	0.00	0.01*
	(0.00)	(0.00)
Female	0.33***	0.66***
	(0.09)	(0.10)
Single	0.19	0.31**
	(0.12)	(0.12)
nrgups	– 0.01	0.06
	(0.04)	(0.07)
nrkids	– 0.06	– 0.06
	(0.08)	(0.08)
Living space	– 0.00	– 0.00
	(0.00)	(0.00)
Infected	– 0.28	0.02
	(0.40)	(0.40)
Can protect oneself	– 0.20	0.31**
	(0.13)	(0.13)
Sector	– 0.17	– 0.08
	(0.13)	(0.11)
Locus of control	0.60***	0.41***
	(0.08)	(0.07)
Job neg. affected	0.31**	0.50***
	(0.14)	(0.15)
Self-employed	0.48**	0.95***
	(0.21)	(0.22)
Incidence	0.00	0.00
	(0.00)	(0.00)
Constant	– 3.02***	– 4.15***
	(0.43)	(0.47)
Observations	1321	1327
R-squared	0.29	0.31

Robust standard errors in parentheses

***$$p<0.01$$, **$$p<0.05$$, *$$p<0.1$$

The coefficient on the interaction between reactance and posterior lethality beliefs is negative and highly significant.^6^ This is (suggestive) evidence that the effect of reactance on the loss in life satisfaction seems to be driven mainly by those people in Treatment $T_{1}$ whose beliefs about approximate mortality have been sufficiently “corrected” by the information video shown in the beginning. A comparison of the death rate beliefs between treatment group $T_{0}$ (no information provided) and treatment groups $T_{1}$ shows that the information provided in treatment $T_{1}$ had indeed an information effect on respondents (see supplementary material).

The finding that the effect of reactance on the loss in subjective life satisfaction is in fact present in the weaker information treatment $T_{1}$ as well, but mainly for those respondents who have updated their beliefs about average mortality of Covid-19 (see Table 2), offers further support for our conjecture that what affects the loss in subjective life satisfaction is not reactance per se: even individuals with a high reactance index do not suffer more than others from the curtailment of personal freedom if they think that the imposed measures serve a vital purpose—namely the containment of a dangerous and very lethal virus. However, once they realize that lethality rates are not as high as they believed, those highly reactant individuals are the ones who report a larger loss in life satisfaction than the rest.

## Discussion and Conclusion

Our analysis was motivated by the Covid-19-related curtailment of personal freedoms and by the question how this might reduce subjective life satisfaction. Making use of heterogeneity in individuals’ preferences for such freedoms and of randomized information concerning the danger of Covid-19, we find a direct effect of the curtailment of freedom rights on life satisfaction, which is strengthened if people are informed about Covid-19 lethality (which is overestimated on average among uninformed respondents), in particular for respondents with high psychological reactance.

We measure an overall loss in life satisfaction in the pandemic crisis of 2020. This overall reduction in life satisfaction between the beginning of the year and April can reflect many different aspects of the crisis in addition to feelings about being deprived of behavioral freedoms. Among them are concerns about health risks for oneself or for beloved others, worries about an uncertain future, a gloomy financial perspective and looming unemployment. The multivariate analysis shows that several of these aspects have played a role and contributed to the loss in life satisfaction. The empirical estimates show strong effects for employees who fear negative consequences for their job or employment loss. Even stronger effects apply for the self-employed and entrepreneurs. Many self-employed had to fear that they would not only lose the returns from hard work and entrepreneurial spirit, but also the foundations of their economic existence. And entrepreneurs had to fear that their companies, often built up over generations, would not survive and/or that they themselves would go into personal insolvency with their company.

Our data also shows that respondents who reach a high score on the psychological reactance scale suffer a higher than average loss in life satisfaction after learning that the lethality rate among people infected with the Covid-19 virus is (on average) lower than they had initially expected. This holds especially for the treatment group which was informed about the age distribution of death rates (Treatment $T_{2}$) and not only about the overall average death rate (Treatment $T_{1}$). After having learned that the death risk for themselves and for their families and friends was much lower than they had initially believed, other aspects of the crisis than health aspects might have seemed more important to them and the justification for the restrictions of their personal freedom might have eroded in their eyes.

A deeper look into the data and the experimental design by making use of the treatment effects of random assignment of respondents to three different information treatments, hints at a complex mechanism that explains this effect. ‘Reactance’ alone, understood as an aversion to regulations and impositions, does not serve perfectly well as an indicator for the life satisfaction effects of the curtailment of freedom rights. The life satisfaction reducing effects of high reactance emerge in fact only among the set of respondents who were subject to an information treatment. This information treatment gave them an opportunity to update their beliefs about the lethality of Covid-19. Given their prior beliefs, on average, respondents had to adjust their beliefs about lethality downwards. Respondents who receive information concerning the lethality of Covid-19 learn that the disease is less dangerous than they believed before. This updating might lead to immediate revisions in their views about the necessity and appropriateness of the freedom-curtailing policy measures. Combined with a strong preference for personal freedom, this might make them less happy if personal freedom rights are curtailed.

Finally, note that the information we provided was concerning death rates in China. One might of course object to using this data as informative about how lethal the virus was expected to be in Germany (which would be the relevant information for our survey participants in terms of assessing the danger of the virus in Germany and the necessity of the curtailment measures). We decided to use the Chinese data in our information treatments because at the time the study was run there was no statistically solid information about death rates in Germany, where the first wave of infections had just started (while in Wuhan the first wave of infections had already subsided). However, as long as our participants regarded the death rates in China as being (at least to some extent) informative about the danger of the virus in Germany, and as long as their beliefs about lethality in China were not completely disconnected from their beliefs about expected mortality in Germany, we can safely assume that our information treatments have lowered the perceived danger of the virus on average.

Overall, we interpret our results as being in line with the general hypothesis that the curtailment of behavioral freedom can go along with a reduction in life satisfaction, in particular for individuals who are averse to restrictions imposed from outside. However, we find that whether or not the curtailment of freedom triggers a loss, as well as the size of the loss, are not just a function of individuals’ reactance. The loss depends in more intricate ways on what respondents know about the situation in which such freedom restricting measures are taken and whether these restrictions are ‘justified’ in their eyes.

## Supplementary Information

Below is the link to the electronic supplementary material.

Supplementary material 1 (pdf 2540 KB)

## Footnotes

1 This concept is well established. See surveys by Frey and Stutzer (2002), Helliwell and Putnam (2004), Clark et al. (2008), Diener (2012), Clark (2018), and Diener et al. (2018).

2 See e.g. Inglehart and Klingemann (2000), Veenhoven (2000), Frey and Stutzer (2002), Inglehart et al. (2008), Gehring (2013), Graafland and Compen (2015), Spruk and Kesšeljević (2016), Jackson (2017) and Nakazato et al. (2017).

3 At the beginning of the survey we asked participants to rate their (remembered) life satisfaction in January (2020) on a scale from 1 to 10. Later on in the survey, after the information treatments, we asked them to rate their *current* life satisfaction in the same way. The exact wording of the questions (translated to English) can be found in the supplementary material.

4 We include this control variable to account for the fact that respondents might report different losses of life satisfaction depending on their initial levels.

5 Due to the discrete nature of our outcome variable (which can take integer values from – 9 to 9), this would be the most appropriate regression model in our case, but the estimated coefficients are difficult to interpret in a meaningful way (without calculating marginal effects). In addition, as the outcome variable, despite being of a discrete nature, can take many values, we regard the OLS method as justifiable in this case. A comparison of our regression results shows that all the coefficients which are significant with OLS turn out to remain significant (and have the same sign) in an ordered probit regression.

6 A similar analysis can be performed for treatment group $T_{2}$. However, there we find that, though the coefficient on the interaction term is also negative, it is not significant at conventional levels (see column (2) of Table 2). This suggests that, unlike in $T_{1}$, all reactant people in $T_{2}$ are affected equally (and more than respondents in $T_{1}$), irrespective of how much they “update” their lethality beliefs. We don’t have a clear explanation for why correct updating does not seem to matter for the strength of the effect in $T_{2}$, or—put differently—why the effect of $T_{2}$ is strong irrespective of the effect on ex-post beliefs. Note however that our measure of “correct updating” (namely checking how well respondents are able to recall average lethality) might not be suitable (or measure what we want it to measure) in this case, because we provide lethality rates for all age classes in $T_{2}$, but do not mention average lethality explicitly. Respondents might thus have a hard time assessing average lethality given the provided information. Hence, even respondents who are not stating a value of average lethality close to the “correct” number might have lowered their assessment of the lethality (and thus the expected danger) of the virus.

## Declarations

### Pre-registration

AEA RCT Registry nr. AEARCTR-0005703.
