# Supplementary material for: Curtailment of Civil Liberties and Subjective Life Satisfaction
Source: J Happiness Stud. 2021 Dec 22;23(5):2157–70. doi: 10.1007/s10902-021-00491-1 (PMC8692827; doi:10.1007/s10902-021-00491-1)
Supplement: Supplementary file 1 — Supplementary material 1 (pdf 2540 KB) [file 10902_2021_491_MOESM1_ESM.pdf]

Curtailment of Civil Liberties and Subjective Life Satisfaction: Supplementary material

Lisa Windsteiger[*], Michael Ahlheim[†], and Kai A. Konrad[‡]

[*]Max Planck Institute for Tax Law and Public Finance, Marstallplatz 1, 80539 Munich, Germany. Email: lisa.windsteiger@tax.mpg.de.

[†]Institute of Economics (520F), University of Hohenheim, 70599 Stuttgart, Germany. Email: ahlheim@uni-hohenheim.de.

[‡]Max Planck Institute for Tax Law and Public Finance, Marstallplatz 1, 80539 Munich, Germany. Email: kai.konrad@tax.mpg.de.

1 Factor analysis to obtain an index for psychological reactance

We use 14 questions designed to elicit psychological reactance (see Hong and Page (1989)). The 14 questions we used were asked as follows in our questionnaire (see Section 5 for the full questionnaire):
To what extent do you agree with the following statements?
R1: *Regulations trigger a sense of resistance in me. (Strongly agree - Agree more or less - Neither agree nor disagree - Rather not agree - Do not agree at all)*
R2: *I find contradicting others stimulating. (Strongly agree - Agree more or less - Neither agree nor disagree - Rather not agree - Do not agree at all)*
R3: *When something is prohibited, I usually think, "That's exactly what I am going to do". (Strongly agree - Agree more or less - Neither agree nor disagree - Rather not agree - Do not agree at all)*
R4: *The thought of being dependent on others aggravates me. (Strongly agree - Agree more or less - Neither agree nor disagree - Rather not agree - Do not agree at all)*
R5: *I consider advice from others to be an intrusion. (Strongly agree - Agree more or less - Neither agree nor disagree - Rather not agree - Do not agree at all)*
R6: *I become frustrated when I am unable to make free and independent decisions. (Strongly agree - Agree more or less - Neither agree nor disagree - Rather not agree - Do not agree at all)*
R7: *It irritates me when someone points out things which are obvious to me. (Strongly agree - Agree more or less - Neither agree nor disagree - Rather not agree - Do not agree at all)*
R8: *I become angry when my freedom of choice is restricted. (Strongly agree - Agree more or less - Neither agree nor disagree - Rather not agree - Do not agree at all)*
R9: *Advice and recommendations usually induce me to do just the opposite. (Strongly agree - Agree more or less - Neither agree nor disagree - Rather not agree - Do not agree at all)*
R10: *I am contended only when I am acting of my own free will. (Strongly agree - Agree more or less - Neither agree nor disagree - Rather not agree - Do not agree at all)*
R11: *I resist the attempts of others to influence me. (Strongly agree - Agree more or*

Table 1: Eigenvalues from Factor Analysis for Reactance.

	Factor1	Factor2	Factor3	Factor4	Factor5	Factor6
Eigenvalues	4.81	.96	.23	.13	.068	-.01

less - Neither agree nor disagree - Rather not agree - Do not agree at all)
R12: *It makes me angry when another person is held up as a role model for me to follow. (Strongly agree - Agree more or less - Neither agree nor disagree - Rather not agree - Do not agree at all)*
R13: *When someone forces me to do something, I feel like doing the opposite. (Strongly agree - Agree more or less - Neither agree nor disagree - Rather not agree - Do not agree at all)*
R14: *It disappoints me to see others submitting to society's standards and rules. (Strongly agree - Agree more or less - Neither agree nor disagree - Rather not agree - Do not agree at all)*

We perform a standard exploratory factor analysis. Five factors with positive Eigenvalues were identified. The most commonly used method to decide how many factors to retain from factor analysis is the Kaiser criterion, which recommends retaining all factors with Eigenvalues greater than 1 (and only those), as any additional factors corresponding to smaller Eigenvalues would only account for trivial variance (see Kaiser (1960)). From Table 1 we can see that only one factor emerges with corresponding Eigenvalue larger than unity. We thus decided to retain a single factor to capture reactance. This factor accounts for more than 90% of the total variance in all the variables. The factor loadings for this factor are given in Table 2.

Table 2: Factor Loadings from Factor Analysis for Reactance, 1 Factor

	Factor1
R1	.673615
R2	.5155176
R3	.5693014
R4	.5504937
R5	.5890258
R6	.6199371
R7	.4689748
R8	.5787915
R9	.6733165
R10	.5995053
R11	.538607
R12	.5722273
R13	.6506077
R14	.5639372

2 Factor analysis to obtain an index for Locus of Control

We use four questions designed to elicit locus of control (see Kovaleva et al., 2012) to conduct an exploratory factor analysis. We find one factor with Eigenvalue larger than 1 (see Table 3) and thus retain one factor to capture locus of control (see Kaiser (1960)). The factor loadings are given by Table 4. The four questions we used were asked as follows in our questionnaire (see Section 5 for the full questionnaire):
The following statements may more or less apply to you. For each statement, please include to what extent this applies to you personally:
L1: *I'm in charge of my own life. (Fully true - More likely to be true - Neither true nor false - Rather not true - Not true at all)*
L2: *If I make an effort, I will succeed. (Fully true - More likely to be true - Neither true nor false - Rather not true - Not true at all)*
L3: *Whether privately or professionally: My life is largely determined by others. (Fully true - More likely to be true - Neither true nor false - Rather not true - Not true at all)*
L4: *My plans are often thwarted by fate. (Fully true - More likely to be true - Neither true nor false - Rather not true - Not true at all)*

Table 3: Eigenvalues from Factor Analysis for Locus of Control.

	Factor1	Factor2	Factor3	Factor4
Eigenvalues	1.228	.163	-.189	-.238

Table 4: Factor Loadings from Factor Analysis for Locus of Control

	Factor1
L1	.582
L2	.595
L3	-.577
L4	-.45

3 Additional regression tables

Table 5: Ordered probit regression of the loss in subjective life satisfaction on reactance and controls. Specification (1) denotes the regression over the whole sample, (2) (3) and (4) the split sample regressions looking at each treatment group separately, and (5) is the regression over the whole sample including interactions between treatment group and reactance.

VARIABLES	(1)<br>wholesample<br>swb loss	(2)<br>T_0<br>swb loss	(3)<br>T_1<br>swb loss	(4)<br>T_2<br>swb loss	(5)<br>interactions<br>swb loss
reactance	0.09***	0.04	0.08**	0.17***	0.05
	(0.02)	(0.03)	(0.04)	(0.03)	(0.03)
1.Treatment	0.01				0.01
	(0.04)				(0.04)
2.Treatment	0.08*				0.08*
	(0.04)				(0.04)
1.Treatment*reactance					0.03
					(0.05)
2.Treatment*reactance					0.11**
					(0.05)
swb old	0.35***	0.35***	0.37***	0.34***	0.35***
	(0.01)	(0.02)	(0.02)	(0.02)	(0.01)
hhincome	-0.00	-0.00	0.00	0.00	-0.00
	(0.00)	(0.00)	(0.00)	(0.00)	(0.00)
Age	0.00*	0.00	0.00	0.00*	0.00*
	(0.00)	(0.00)	(0.00)	(0.00)	(0.00)
Female	0.23***	0.13**	0.22***	0.36***	0.23***
	(0.04)	(0.06)	(0.06)	(0.06)	(0.04)
Single	0.10**	0.01	0.09	0.19***	0.10**
	(0.04)	(0.07)	(0.07)	(0.07)	(0.04)
nrgups	0.01	-0.01	0.01	0.03	0.01
	(0.02)	(0.03)	(0.03)	(0.04)	(0.02)
nrkids	0.01	0.07**	-0.04	-0.02	0.01
	(0.03)	(0.03)	(0.06)	(0.05)	(0.03)
living space	-0.00**	-0.00	-0.00	-0.00	-0.00**
	(0.00)	(0.00)	(0.00)	(0.00)	(0.00)
infected	-0.17	-0.15	-0.40	0.07	-0.17
	(0.16)	(0.30)	(0.25)	(0.22)	(0.16)
can protect oneself	-0.02	-0.14*	-0.14*	0.20**	-0.02
	(0.05)	(0.07)	(0.08)	(0.08)	(0.05)
sector	-0.10**	-0.10	-0.13*	-0.05	-0.10**
	(0.04)	(0.08)	(0.08)	(0.07)	(0.04)
locus of control	0.31***	0.34***	0.37***	0.24***	0.31***
	(0.03)	(0.04)	(0.05)	(0.04)	(0.03)
job neg. affected	0.19***	0.20**	0.19**	0.25***	0.20***
	(0.05)	(0.08)	(0.08)	(0.09)	(0.05)
self-employed	0.41***	0.47***	0.30**	0.48***	0.41***
	(0.09)	(0.18)	(0.14)	(0.13)	(0.09)
incidence	-0.00	-0.00	0.00	0.00	-0.00
	(0.00)	(0.00)	(0.00)	(0.00)	(0.00)
Observations	3,996	1,348	1,321	1,327	3,996

Robust standard errors in parentheses
*** p<0.01, ** p<0.05, * p<0.1

Control variables included in the regression are: equivalized household income, age, gender, marital status, number of grown-ups in the household, number of children in the household, equivalized living space, a dummy for whether the respondent has been infected with Covid-19, a dummy for whether the respondent (thinks she) can take measures to protect herself from infection also once the lockdown is lifted, a dummy for whether the respondent works in a sector with high work load at the moment (health, education, groceries or police), locus of control, a dummy for whether the job is negatively affected, a dummy for whether the respondent has his/her own company or shop, the 7-day-incidence rate of Covid-19 cases in the respondent's region at the time of the survey, and the state in which the respondent lives (not shown in regression table).

4 Lethality beliefs

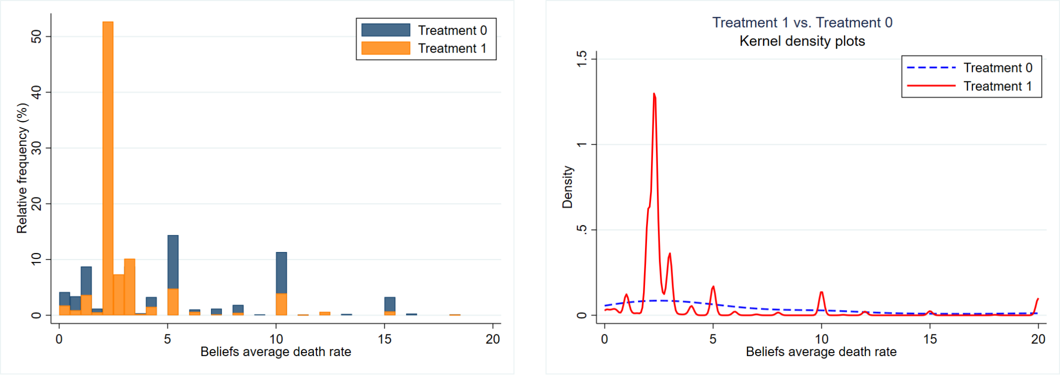

(a) Relative frequency of beliefs about average lethality, T_1 compared to T_0

(b) Gaussian kernel density of beliefs about average lethality, T_1 compared to T_0

Figure 1: Comparison of beliefs about average lethality in T_1 compared to T_0. No information about lethality was provided in T_0, whereas in T_1 participants were informed that average lethality in the Wuhan area had been 2.3%.

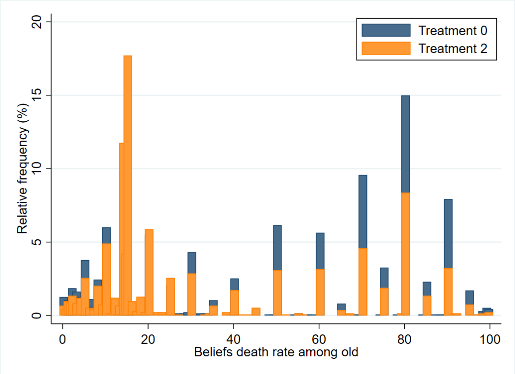

(a) Relative frequency of beliefs about lethality among the old (+80 years old), T_2 compared to T_0

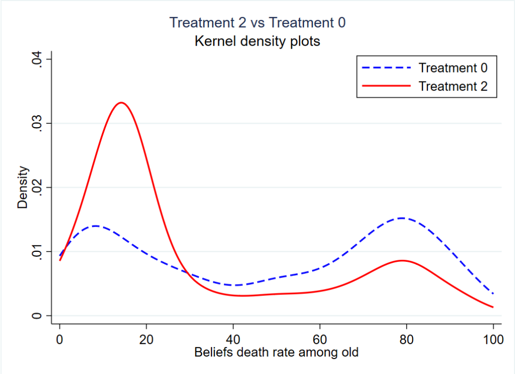

(b) Gaussian kernel density of beliefs about lethality among the old (+80 years old), T_2 compared to T_0

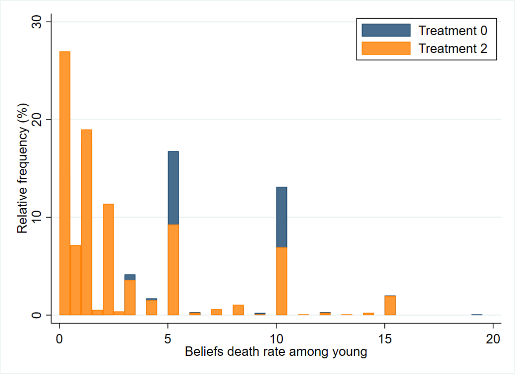

(c) Relative frequency of beliefs about lethality among the young (20-29 years old), T_2 compared to T_0

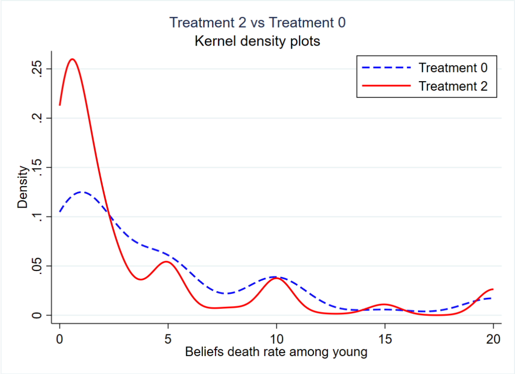

(d) Gaussian kernel density of beliefs about lethality among the young (20-29 years old), T_2 compared to T_0

Figure 2: Comparison of beliefs about average lethality in T_2 compared to T_0. No information about lethality was provided in T_0. In T_2 participants were not informed about average lethality either, but they were informed about lethality rates by age group in the Wuhan area (ranging from 0.2% for the young to 14.8% for people over 80).

5 Questionnaire

(English translation of the) Survey on
The Impact of the Corona Crisis on Private Households

We are a group of non-partisan scientists at an institute of the Max Planck Society and at the University of Hohenheim. We would like to ask you about your personal opinions regarding the restrictions on private and public life that the German federal government, in conjunction with the state governments, took on 22nd March in response to the corona crisis and which have the declared aim of reducing the speed at which corona virus infections spread.

It is important for the quality and effectiveness of the study that you answer honestly and read the questions very carefully before you answer. It is also important that you fill in the whole questionnaire after you have started the survey. This survey should (on average) take about 15 minutes.

Please note: Your participation in this study is completely voluntary. We will only receive your answers in anonymous form. This anonymized data will be stored securely and used for scientific purposes only.

○ Yes, I would like to participate in this study.

○ No, I would not like to participate in this study.

Thanks a lot for your participation!

____

**1.**	Are you male or female?	○ Female<br>○ Male
**2.**	How old are you?	_________
**3.**	What is your marital status?	○ Single (never married / widowed / separated / divorced)<br>○ Married / registered partnership / living together with partner
**4.**	How many people live in your household? (yourself included)	______ adults (and children over 14 years of age)<br>______ children (under 14 years)
**5.**	What was the average monthly disposable income of all persons living in your household in the last 12 months?	○ Less than 2000 €<br>○ Between 2000 and under 3000 €<br>○ Between 3000 and under 4000 €<br>○ Between 4000 and under 5000 €<br>○ Between 5000 and under 6000 €<br>○ Between 6000 and under 7000 €<br>○ 7000 € or more
**6.**	Please select your federal state and your region/city: (Drop-down)	
**7.**	Please try to remember your life situation at the beginning of this year. How satisfied were you then, all in all, with your life? (1 = not satisfied at all, 10 = completely satisfied)<br>○ 1 ○ 2 ○ 3 ○ 4 ○ 5 ○ 6 ○ 7 ○ 8 ○ 9 ○ 10	

We will now ask you for your opinion on the measures to contain the Corona virus. Please review the following information before proceeding:

Information Treatment Block: At this stage, either Treatment 0, Treatment 1 or Treatment 2 were randomly displayed to the respondents. For a detailed description of our information treatments, see Section "Information treatments"

In March, the Federal Government and the federal states adopted measures to stem the corona epidemic, which restrict economic and social life. In addition to contact or exit restrictions on residence and movement outside the home, these include other restrictions including the following:

(A) The closure of shops, shopping centres and services where physical contact is unavoidable (e.g. hairdressers, beauty salons, etc.)
(B) The closure or restriction of the running of many leisure facilities (restaurants, bars, clubs, discotheques, recreational sports facilities, theatres, etc.)
(C) The closure of facilities such as day-care centres, schools, etc. where underage children are supervised
(D) Strict visitation bans for retirement homes, hospitals, etc.
(E) Closure of many offices and authorities, universities etc. to the public

**8.**	Do you think that the restrictions valid until April 20 are correct in their entirety?	○ Exactly right<br>○ Too radical<br>○ Too tentative		
**9.**	Are you in favour of the measures being continued for another 4 weeks after April 20?	○ Yes<br>○ No<br>○ Cannot decide		
**10.**	Who, in your opinion, is more likely to be harmed by restrictive measures	○ Rather the younger	○ Rather the elderly	○ Both alike
		○ Rather the poorer	○ Rather the richer	○ Both alike
		○ Rather men	○ Rather women	○ Both alike
**11.**	How much do you agree with the statement: "I am prepared to temporarily give up some of my liberties if it helps to slow down the spread of the virus"?	○ Strongly agree<br>○ Agree more or less<br>○ Neither agree nor disagree<br>○ Rather not agree<br>○ Do not agree at all		
**12.**	How threatening do you consider the virus for yourself personally, e.g. in comparison to the average of society?	○ Rather higher	○ Rather lower	○ About the same
**13.**	Do you have relatives who are particularly at risk of infection by the virus?	○ Yes	○ No	○ I don't know
**14.**	Are or were you infected with the virus?	○ Yes	○ No	○ I have not been tested yet

15. As already mentioned, the Federal Government and the Länder have adopted measures to contain the corona epidemic, which restrict economic and social life. In addition to contact or exit restrictions on residence and travel outside the home, these include other restrictions such as the following: (A) The closure of shops, shopping centres and service providers where physical proximity is unavoidable (e.g. hairdressers, beauty salons, etc.) (B) The closure or restriction of the operation of many leisure facilities (restaurants, bars, clubs, discotheques, leisure sports facilities, theatres, etc.) (C) The closure of facilities such as day-care centres, schools, etc. where underage children are supervised. (D) Prohibiting visits to retirement homes, hospitals, etc. (E) The closure of many offices and authorities, universities etc. to the public.

Assume that you could lift any of the above measures with immediate effect: Which of the five measures (A) to (E) would you end first? Which one would you end second? Please rank the measures according to the urgency with which you would like to repeal them. The measure that you would most urgently like to repeal will be at the top, the second below, etc. You can move the individual measures (A) to (E) by clicking on them and dragging them to the place where you would like them to be (Drag & Drop).

First:

Second:

Third:

Fourth:

Fifth:

16. You would like to terminate measure [*insert respondent's choice*] first. What would it be worth to you if this restrictive measure were to be dropped - what amount of money would you give up per month next year (i.e. a total of 12 times) in return?

-------- Euro per month

17. What amount of money would you forego per month next year (i.e. a total of 12 times) for lifting all measures?

--------- Euro per month

18. Now we would like to know how you feel today. How satisfied are you in the present situation, all in all, with your life? (1 = not satisfied at all, 10 = completely satisfied)

o 1 o 2 o 3 o 4 o 5 o 6 o 7 o 8 o 9 o 10

To what extent do the following statements about the effects of the restrictive measures apply to you personally?

	Fully true	More likely to be true	Rather not true	Not true at all	No statement
**19.** Living together in the household has been difficult in recent weeks.	○	○	○	○	○
**20.** I was worried about my health.	○	○	○	○	○
**21.** Boredom was a problem.	○	○	○	○	○
**22.** I was feeling lonely.	○	○	○	○	○
**23.** There were supply shortages.	○	○	○	○	○
**24.** I fear great negative financial consequences in the future.	○	○	○	○	○
**25.** Do you think that the current situation in your personal environment will lead to assaults and domestic violence?			○ Yes	○ No	○ Don't know.

How much do you agree with the following statements?

	Strongly agree	Agree more or less	Neither agree nor disagree	Rather not agree	Do not agree at all
**26.** I trust that the government is doing the right thing.	○	○	○	○	○
**27.** I trust that the government is doing the right thing in health policy matters.	○	○	○	○	○
**28.** I am confident that the government is doing the right thing in fiscal and economic policy matters.	○	○	○	○	○

The following statements may more or less apply to you. For each statement, please include to what extent this applies to you personally.

	Fully true	More likely to be true	Neither true nor false	Rather not true	Not true at all
**29.** I'm in charge of my own life.	○	○	○	○	○
**30.** If I make an effort, I will succeed.	○	○	○	○	○
**31.** Whether privately or professionally: My life is largely determined by others.	○	○	○	○	○
**32.** My plans are often thwarted by fate.	○	○	○	○	○

To what extent do you agree with the following statements?

		Strongly agree	Agree more or less	Neither agree nor disagree	Rather not agree	Do not agree at all
**33.**	Regulations trigger a sense of resistance in me.	○	○	○	○	○
**34.**	I find contradicting others stimulating.	○	○	○	○	○
**35.**	When something is prohibited, I usually think, "That's exactly what I am going to do".	○	○	○	○	○
**36.**	The thought of being dependent on others aggravates me.	○	○	○	○	○
**37.**	I consider advice from others to be an intrusion.	○	○	○	○	○
**38.**	I become frustrated when I am unable to make free and independent decisions.	○	○	○	○	○
**39.**	It irritates me when someone points out things which are obvious to me.	○	○	○	○	○
**40.**	I become angry when my freedom of choice is restricted.	○	○	○	○	○
**41.**	Advice and recommendations usually induce me to do just the opposite.	○	○	○	○	○
**42.**	I am contended only when I am acting of my own free will.	○	○	○	○	○
**43.**	I resist the attempts of others to influence me.	○	○	○	○	○
**44.**	It makes me angry when another person is held up as a role model for me to follow.	○	○	○	○	○
**45.**	When someone forces me to do something, I feel like doing the opposite.	○	○	○	○	○
**46.**	It disappoints me to see others submitting to society's standards and rules.	○	○	○	○	○

Now we have some general questions for you:

**47.**	What is your highest school leaving certificate (incl. college or university degree)?	○ I did not finish school.<br>○ Secondary school or comparable qualification<br>○ Baccalaureate or equivalent<br>○ Completed university or technical college studies
**48.**	Which employment group do you currently belong to?	○ I am employed in the private sector.<br>○ I am employed in the public sector.<br>○ I am self-employed and have no employees.<br>○ I am self-employed and have one or more employees.<br>○ I am unemployed.<br>○ I go to school or study.

		○ I am retired.<br>○ I am not currently employed and am not looking for work.<br>○ I do not belong to any of these groups.
49.	If you are self-employed: Is your company or business affected by the restrictions?	○ Lower revenues.<br>○ Higher revenues.<br>○ No amendments.<br>○ I had to shut down operations completely.<br>○ No statement.
50.	If you are employed as an employee: How is your workplace affected by the restrictions?	○ I had to switch to short-time work.<br>○ My job is threatened by the crisis.<br>○ There have been no effects so far.<br>○ No statement.
51.	Do you work in one of the following sectors?	○ Health care (hospital, doctor's surgery, nursing, pharmacy)<br>○ Food supply<br>○ Education<br>○ Police /Fire department<br>○ I don't work in any of these sectors.

Now we would like to ask you about your current living situation:

52.	Do you live alone or together with other people?	○ Alone<br>○ With my family<br>○ In a flatshare<br>○ In another form of housing (e.g. in a home, sublet, etc.)
53.	Do you currently live in an apartment or a whole house?	○ In an apartment<br>○ In a house<br>○ In a room (e.g. in a flatshare or home)
54.	Do you live in property or on rent?	○ Own property<br>○ On rent<br>○ Rent-free (e.g. with my parents)
55.	Do you have an outdoor area?	○ Balcony or Loggia<br>○ Garden<br>○ No outdoor area
56.	How many rooms does your living area have?	Nr of rooms:
57.	How many square meters does your living area cover?	Square meters:

		Yes	No	No statement
**58.**	Do you have the possibility, even after the restrictive measures have been lifted, to lead your life for the most part in such a way that you keep your risk of infection as low as possible?	O	O	O
**59.**	Were you born in Germany?	O	O	O
**60.**	Were both of your parents born in Germany?	O	O	O

**61.**	It is difficult to assess how dangerous a virus is during a pandemic.<br>A study for China has analysed what proportion of people infected with corona have died during the course of the disease. How high do you estimate this proportion?	_______ %
**62.**	How high do you estimate this proportion for people older than 80?	_______ %
**63.**	How high do you estimate this proportion for people between 20 and 29 years of age?	_______ %

Thank you very much for your participation!

6 Information treatments

We randomly assigned the respondents to one of three information treatments. To provide a clear baseline, we included a "neutral treatment" T_0 in which respondents read a neutral sentence only referring to the fact that the government introduced measures to combat the spread of Covid-19. The other treatments (also include that sentence and) vary whether respondents were informed about:

- the *average* estimated lethality rate of Covid-19 (T_1),
- the lethality *by age group*, indicating a clear increase of fatalities for higher age groups (T_2),

The experimental information was conveyed to the respondents by means of video clips which they visualized in the course of the survey. Stills of the clips are provided in Figures 3 to 5 (English translation below each still).

Each of the videos began with a statement referring to the fact that the government introduced measures to combat the spread of Covid-19. Only the video for the baseline treatment then continued immediately with an invitation for the respondent to proceed with the survey. The other videos informed our respondents of the estimated lethality rate of Covid-19 as described above.

Treatment 0 The information provided in Treatment T_0 only generically referred to the fact that the government introduced measures to combat the spread of Covid-19 without mentioning estimated lethality rates.

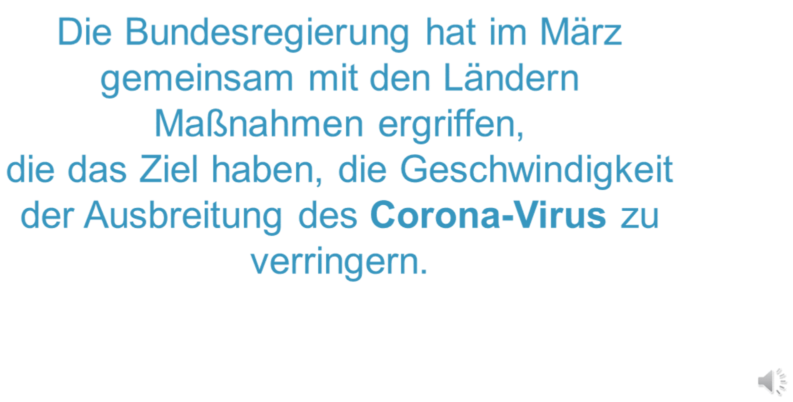

Figure 3: Information provided in T_0: *In March the German government together with the federal states announced restrictions aimed at reducing the speed of the spread of the corona virus.*

Treatment 1 Figure 4 displays the information given to the respondents assigned to Treatment T_1. This group of respondents received information about the fact that the estimated average lethality rate of Covid-19 in China was 2.3%.

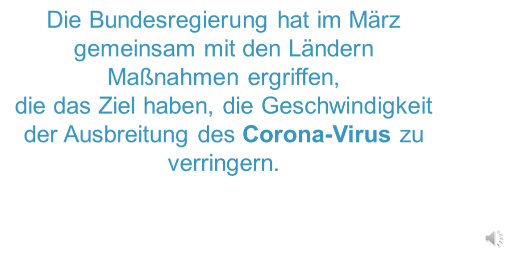

(a) *In March the German government together with the federal states announced restrictions aimed at reducing the speed of the spread of the corona virus.*

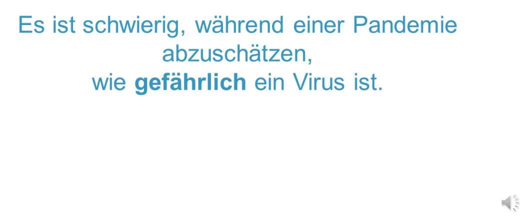

(b) *It is difficult to assess how dangerous a virus is during a pandemic.*

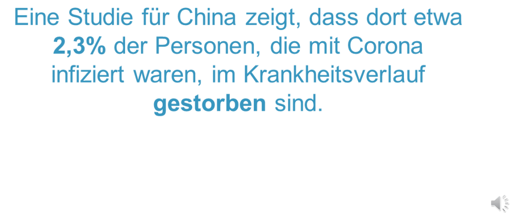

(c) *A study for China shows that approximately 2.3% of those infected with the virus died during the course of the illness.*

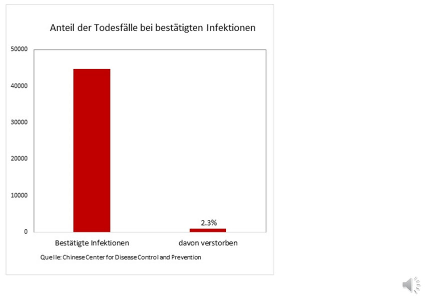

(d)

Figure 4: Information provided in T_1

Treatment 2 Figure 5 displays the information given to the respondents assigned to Treatment T_2. This group of respondents received information about the fact that the estimated lethality rate of Covid-19 in China was increasing in age.

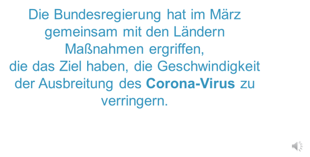

(a) *In March the German government together with the federal states announced restrictions aimed at reducing the speed of the spread of the corona virus.*

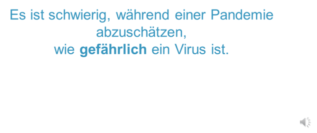

(b) *It is difficult to assess how dangerous a virus is during a pandemic.*

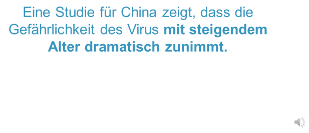

(c) *A study for China shows that the danger of the virus increases dramatically with age.*

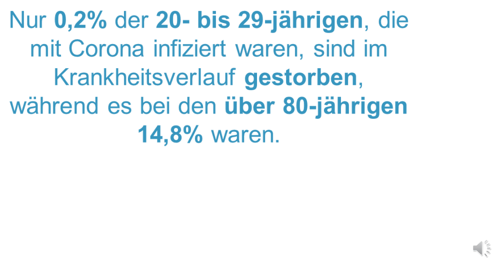

(d) *Only 0.2% of 20-29 year olds infected with corona died during the course of the illness, compared to 14.8% of those over 80.*

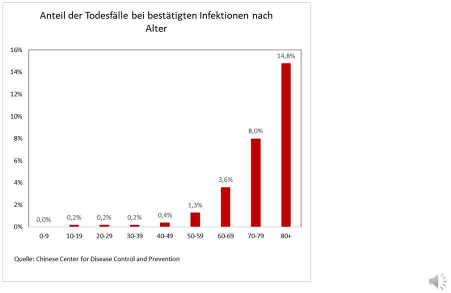

(e)

Figure 5: Information provided in T_2
